# Interventions to reduce inequalities in health and early child development in Europe from a qualitative perspective

**DOI:** 10.1186/s12939-017-0584-0

**Published:** 2017-05-25

**Authors:** Joana Morrison, Hynek Pikhart, Peter Goldblatt

**Affiliations:** 1 Department of Epidemiology and Public Health, University College London, 1-19 Torrington Place, London, WC1E 6BT UK; 2 UCL Institute of Health Equity, Department of Epidemiology and Public Health, London, UK

**Keywords:** Child development, Early intervention, Socioeconomic factors, Poverty

## Abstract

**Background:**

Early childhood is a critical stage of development. Inequalities in experiences affect children’s wellbeing and determine their development. Early years interventions focusing on children and their parents may help address inequalities during this critical period. Understanding the experiences and perceptions of parents receiving early years programmes and staff providing these may help service development and delivery. The objective of this study was to describe staff and parents’ accounts of how early childhood programmes in different European country contexts improved child development.

**Methods:**

Five early years programmes were selected using pre-set criteria out of ten proposed ones. Twenty-five individual interviews and six focus groups were carried out with staff running interventions and with users, children and their families in different EU countries. Investigations of the studies were carried out using qualitative research methods. Data were collected by collaborating partner institutions included in the project.

**Results:**

Participants described programmes which aimed to provide activities to stimulate children’s learning through structured play and which provided support and assistance for parents. In these, parents were actively involved in activities. Parents and staff referred to establishing long-term trust based relationships as a key element for programmes to improve parents’ self-esteem and reduce their stress levels which in turn helped improve their children’s development.

**Conclusions:**

Programmes described by staff as being successful, delivered services tailored to parents and their children. Adapting to and understanding the families’ circumstances and involving parents was seen by staff as important. Staff also described establishing trust based relationships as a key enabler in programme delivery; their perceptions were that parents should be empowered to develop their own capacities thus strengthening their abilities to assist in their children’s learning, which had a positive effect on children.

## Strengths and limitations

- Data collection was carried out by partners in each country; this enabled gaining access to hard to reach groups and participants. Collaborating partners liaised with expert gatekeepers and stakeholders in the field.
- The analysis was carried out by Joana Morrison however the interviews were carried out by collaborating local researchers in the local language.
- Collaborating with multiple academic and non-academic parties in different regions of Europe enabled collecting rich data on perceptions and beliefs on interventions to improve child development within areas which are less represented in international public health journals.
- A common research protocol and guide was provided to collaborating partners carrying out fieldwork to provide guidance and ensure homogeneity in data collection.
- Collaborating party members carrying out the data collection had different levels of experience regarding qualitative research methods. However they all had degrees in higher education.
- The first and last authors visited the intervention sites and discussed data collection issues with third parties and provided guidance, this was a sustained iterative process.

## Background

A key requirement for healthy early child development (ECD) is a secure relationship with a primary caregiver [1, 2]. Resilience is the capacity to thrive in adverse circumstances [3] and families provide the most important relationships and nurturing environments for enhancing it [4]. Many families that face daily challenges due to their socioeconomic circumstances are not able to create the necessary nurturing environments for their children [4, 5]. Social protection policies, access to appropriate services and sufficient income help families provide a protective environment that fosters children’s resilience [6, 7]. Lifelong inequalities start before birth and accumulate across the lifecourse [8]. An analysis of inequalities across cohorts from 12 European countries, which also forms part of the Drivers project [9], illustrated intergenerational transmission of inequity; poor health was greater amongst children of mothers with low education. Longitudinal birth cohort studies such as these provide data which can help monitor health inequalities and the impact of early years interventions. The Growing Up in Scotland longitudinal birth cohort commissioned by the Scottish Government showed that at age 4, differences in child health outcomes were less evident than those in risk factors. However, negative outcomes may become more apparent in later life [10].

Interrupting intergenerational transmission of inequalities is an important consideration [11]. Early years interventions, designed to reduce inequalities in health and development and their social determinants, may address these [12, 13]. This can be achieved by focusing on actions which give all children the best start in life and are delivered with intensity proportionate to the social needs of children and their families [14–16]. Ensuring the best start in life for children can be fostered by improving outcomes in the different domains of early child development, namely; cognitive, communication and language, social and emotional and physical [5, 17–19]. Interventions are commonly aimed at children, their parents or both. A systematic review of early years interventions carried out within Drivers [18], found that the majority of interventions identified were targeted at reducing social inequalities of children living in deprived areas but not at levelling the social gradient in health [8]. The objective of this study was to identify early years interventions in different European country contexts and assess their effectiveness in reducing inequalities in health and development through action on the social determinants of health [20, 21]. To achieve the study objective, five early years case studies were selected from a number of proposals - using pre-set criteria (see Methods section) - and in-depth investigations were carried out to identify the efficacy, reach and impact on parenting and developmental outcomes of the interventions represented by the studies.

The study formed part of the DRIVERS Project [4, 22] - a three-year research project funded by the European Union 7th Framework Programme - which focused on three of the key drivers to reduce health inequities: early childhood development, fair employment and working conditions, and welfare, income and social protection. It assessed the impact of policies and programmes and provided policy recommendations and advocacy guidance to reduce health inequalities within Europe. Research was a collaborative effort carried out with non-academic institutions: not-for-profit organisations and agencies across Europe. The project was co-ordinated by EuroHealthNet and Eurochild, partnership organisations focussing on health equity and child well-being within Europe.

### Key questions

At an earlier stage of the DRIVERS project, a systematic review was undertaken to identify early years interventions across Europe that were effective in improving health through social determinants. The limited number of studies available in the literature suggested the most effective approaches were those that addressed both child and parenting issues [23].

Against this background, the following research questions were posed in assessing the programmes: a) what were parents’ and staff’s perceptions regarding the aims of the programmes with reference to child development? b) According to parents and staff, how did these services improve child development? c) What were staff and parents knowledge on who were intended beneficiaries of the programmes? d) What were staff and parent’s experiences regarding activities included in the programmes? e) What were staff and parents’ perceptions regarding barriers and enablers for programme implementation?

## Methods

### Method of selection of case studies

A questionnaire was developed to obtain detailed information on relevant interventions being undertaken by Eurochild members or EuroHealthNet partners who volunteered to participate in the study. Ten programmes were put forward. 1) Family Network in Austria; 2) Prolepsis, a programme on food aid in Greece; 3) Sure Start Hungary; 4) a mother-baby programme for teenage mothers in Hungary; 5) a universal home visit service in Hungary; 6) Eager and Able to Learn aimed at young children’s development in Northern Ireland; 7) Toy Box in Northern Ireland; 8) Maternal Centre Iris, aimed at providing shelter for young mothers and children in Romania; 9) a mother’s club in Romania and 10) the Theotokos centre, Romania. The questionnaire was designed to enable the following selection criteria to be applied in choosing interventions to study: a) country coverage: ensuring selected interventions encompassed sufficient range of countries to reflect the different contexts in Europe, b) aimed at children before they entered school, c) potential to reduce inequalities in health through action on social determinants, d) addressed at least one childhood developmental domain, e) helped with parenting skills and or financial or other support, f) had undergone an evaluation or there was a prospect of carrying one out. The interventions described above were scored against these criteria and the following interventions, explained in detail in Table 1, were selected: the Family Network, Sure Start, a universal home visit service, Toy Box and the Theotokos centre.

**Table 1:** Description of selected interventions

1) The Family Network in Austria: a targeted referral service aimed at families in need with children aged 0–2 and to reduce health inequality by supporting early child development within families facing adverse circumstances providing health care and referrals when needed. This was done by ensuring that families in need received specific support by providing counseling and accompanying families.
2) Toybox from Northern Ireland: an intervention aimed at reaching out to Traveller families to enhance the social, educational, emotional, physical, language and cognitive development of children. By supporting and empowering parents to develop their educational skills, parents participated in children’s learning with positive, non-violent parenting. The intervention was delivered in the family home following individualised plans developed with parents. An objective was to establish trust based relationships with parents and encourage them to become involved in community activities as a support mechanism.
3) The Universal Medical Visitor service from Hungary provided comprehensive medical attention based on the child’s developmental needs and rights. Staff described activities to encourage and promote physical development, communication and emotional stimulation, independency, attention, memory, major motor skills and sense of direction.
4) The Theotokos Centre from Romania aimed at providing unemployed and Roma single mothers and their children with child-care support and programmed structured play activities. The centre emphasised on reinforcing mother and child attachment to prevent child abandonment.
5) The Hungarian adaptation of Sure Start was developed to support children and their families to reduce health inequalities in the most deprived regions. The programme aims to reach families from diverse backgrounds to promote integration of disadvantaged and/or minority - mostly Roma - children and their parents into the community. It establishes cooperation with local services focusing on strengthening parenting capacities and providing advice and support for women seeking employment.

### Data collection

Socioeconomic profile indicators of intervention countries were collected from various data sources (see Table 2). Collaborating institutions carried out individual in-depth semi structured interviews and focus groups (see Table 3 for detailed information on informants) following a common research protocol and using a semi structure topic guide developed by Joana Morrison. Both were designed taking into account interviewers’ and third parties’ feedback and these were discussed in various project meetings and with DRIVERS collaborating third party groups. Third party group members involved in carrying out fieldwork had different levels of knowledge regarding qualitative data collection, therefore the toolkit for data collection and interview guides for the interviews and focus groups were discussed at every step of their development. Collaborating third parties tested these and provided feedback to Joana Morrison. Data collection toolkits and topic guides were then amended. This was an iterative process which took place throughout the duration of the study and communication took place via email, field visits by Joana Morrison and co-authors and various teleconferences and Skype calls. The data collection toolkit was based on COREQ criteria for reporting qualitative research [24]. Participants were informed of study aims and principal objectives.

**Table 2:** Socioeconomic profile of the intervention areas

Indicator	UK	Austria	Hungary	Romania	Year of the indicator
Percent of population aged 0–15 years	17.6	14.4	14.5	15.5	2013
Percent of employed population aged 15–64 years who are women	45.1	46.1	45.6	42.4	2013
Unemployment rate	5.1	5	10.3	7.6	2013
Percent of children aged under 18 in poverty	9.8	8.2	9.4	24.9	2010
Percent of children aged 0–5 years living in overcrowded conditions	29	44	80	71.3^a^	2010
Public spending on family services as a percentage of GDP	1.38	0.57	1.16	2.2	2009
Percent of children aged 0–3 years not in formal child care	73	87	92	85	2012

Notes

^a^For Romania, the indicator is percent of children aged under 18 years living in overcrowded conditions

Sources: International Labour Organization [35], Organization for Economic Cooperation and Development [36] and the European Commission [37]

**Table 3:** Participants in interviews and focus groups by programme

Intervention	Focus groups	Individual interviews
Toybox	- One focus group with 10 staff	- Ten individual interviews with carers
Netzwerk Familie	- One focus group with 11 cooperation partners - One group interview with 4 parents	- Two interviews with the heads of Netzwerk Familie at their premises - One interview with the head of child and youth welfare - One interview with the chairman of a paediatrics association
Sure Start Hungary	- Two focus groups with 8 mothers each	- One interview with a Sure Start expert - One interview with a programme manager
Universal health visitor programme Hungary	NA	- One interview with a health visitor from a rural area - One interview with a health visitor from an urban area - One interview with a supervisor
Theotokos centre	- One focus group with four mothers attending the centre	- Four individual interviews with women who had attended the centre - Two individual interviews with staff working at the centre

#### Individual interviews

Collaborating third party institutions which formed part of the DRIVERS consortium identified and interviewed 25 parents, programme managers and key professionals using an interview guide provided to them by the project [22]. Purposive sampling was used. Semi-structured individual interviews were carried out in the local language and lasted at least one hour.

#### Focus groups

Six focus groups were run (see Table 3), using a topic guide. Managers from interventions identified potential participants. Each focus group had approximately 6–10 participants with similar socio-economic backgrounds, age and occupation and lasted approximately 1 h and 45 min. There were 46 participants in total. The moderator guided the discussion partially, following the discussion topic guide provided, developed with the same criteria as the interview guide. The narrator took notes and recorded the sessions. At the end of the session the narrator offered a short summary of the issues discussed so participants could add or rectify information.

#### Processing and analysis of information

Interviews and focus groups transcripts were translated to English by researchers carrying out data collection in collaboration with other members of third party institutions. They sent these to Joana Morrison with comprehensive summaries of the interviews. Joana Morrison carried out a thematic analysis of the interviews and focus groups with the support of the Atlas.Ti qualitative data analysis programme [25]. The interviews were coded using emerging and pre-established codes, based on the research questions and objectives of the study. Codes were then grouped into larger analysis categories. The analysis was an iterative process, Joana Morrison consulted with the researchers which carried out the data collection and provided them with a debrief and finally with preliminary results for validation. Preliminary results were also sent back to each centre and made available to participants for validation and no objections were made.

## Results

Information obtained from interviews and focus groups is provided below in response to questions explored during data collection. The results have been structured following the topics listed in the interview guide. The information within each topic has been grouped according to an emerging outline with the following themes listed below as section headings.

### Staff and parents’ description of programmes and their aims and objectives with regards to early child development

According to respondents, Toybox, Sure Start and the Theotokos centre, programmes were aimed at improving and promoting child development, focusing on children aged 0–4 from disadvantaged backgrounds in a context of low service provision. Parents and staff referred to a context of high unemployment.

*“The unemployment rate is two or sometimes three times higher than the country average and around every third child is living in a family where no one has a job. The education outcomes are poor. Community relations are strong.”* Hungarian health visitor

When asked about the programmes’ objectives and how these were achieved, learning through play was a recurring theme: Toybox and Sure Start parents and staff agreed that the programmes consisted of learning through play to promote child development. Parents explained in which way programmes fostered this method of learning by providing children and parents with guided activities.

*“Dads were happiest when engaging in children’s building and construction play experiences.”* Toybox staff

Staff noted that their role was to support and enable the family to ensure play continued after the project worker left the home. According to staff, the programme also aimed at strengthening parents’ capacities and their relationship with the local community.

*“We have four aims: to ensure the optimal development of the child; establish a good relationship with the parents; strengthen cooperation between the local community.”* Toybox staff

Theotokos staff explained that whilst providing mothers with guidance and counselling, singing and music was part of the program, as well as movement, coordination and outdoor free play with their children.

*“A tells me and shows me a lot of things. In the centre, he learned a lot of good things; he knows a lot of words and many different songs. I like to go out and play with my kids in the park where we sing and we have fun.”* Theotokos centre mother

### Ways in which programmes improved child development

Toybox staff explained that parents were actively involved in activities which enabled establishing long-lasting trust-based relationships. According to staff and parents, this improved parent’s capacities and fostered positive parenting which in turn helped parents assist in their children’s learning and development.

*“Toybox has been a very successful project in engaging Traveller families and supporting the development of Traveller children through play. It is recognised as a successful model in child development especially among disadvantaged groups.”* Toybox staff

As described by staff and parents, Sure Start’s parenting classes, in combination with self - help groups and personal consultations improved self-esteem, job seeking, self-confidence and parent’s relationship with their children. Every child’s development was assessed and monitored.

*“Documentation is used to follow and measure the development of every child. It is filled out every half year and shared with the parents as well.”* Sure Start manager

Health visitors in Hungary explained that outpatient clinics worked in combination with Sure Start Hungary centres providing the opportunity to reach more mothers and children in a community setting. Across interventions, teams included staff members with diverse backgrounds such as psychology, social services, health care and early years. According to staff, it helped provide comprehensive services to parents and their children.

### Programmes’ impact on parenting styles

Staff across the programmes referred to strengthening parenting capacities and providing them with support. This was deemed important for child development, health and overall well-being by parents and staff. The programmes focused many of their activities on achieving these objectives. Parents across programmes identified improvements in one or more of: children’s self-esteem, learning skills, reading and vocabulary.

*“J has become a lot surer of himself; it’s helped with their speech and they play better together.”* Toybox parents

To encourage positive parenting styles, staff filmed parents and their children and viewed it together then discussed with parents ways in which they could address negative parenting styles. Sure Start and Toybox parents referred to feeling supported by non-judgmental staff which improved self-esteem. This resulted in establishing positive bonds with children.

*“The acceptance of the service by families is very important and we now have Toybox visiting different generations of the same family.”* Toybox staff

The Theotokos centre provided counselling and support for mothers at the centre and focused on preventing child abandonment.

*“My youngest daughter comes here where she is very well. The children are clean, they receive attention, someone takes care of them and there are specialized personnel who deal with them.”* Theotokos centre mother

### Barriers and enablers to programme delivery

 Staff referred to conflicts with other service providers and gaining their acceptance. They identified knowledge, expertise and experience within the team as enablers.

*“Services in my area felt we were stepping on their toes and taking over their good work.”* Participant staff

Users were afraid of being reported to child and youth welfare services and staff described this as a barrier to accessing hard to reach families. For *Netzwerk Familie* and Sure Start parents, a relevant barrier was the stigmatising connotation associated with “seeking help” and not coping with the situation themselves. Parents referred to coordinated multi-professional teams as the principal enabler for a successful and comprehensive delivery. Health visitors in Hungary explained that some professionals working on the intervention were family doctors with no child development and well-being training; parents described some health visitors as conservative and prescriptive. Sure Start Hungary staff members were concerned about the programme’s continuity due to funding restraints. The principle enabler described by staff and parents alike referred to other community members, friends or family attending the programme which helped users gain more trust. Most of the Theotokos Centre mothers referred to transport costs and the risk of contracting infections from other children at the centre, as a barrier.

*“Sometimes he gets sick because there are a lot of children in the same place and make each other ill. Three weeks ago he was very ill with bronchitis, conjunctivitis and I took him home.”* Theotokos centre mother

## Discussion

This study illustrates early years initiatives delivered in Romania, Hungary, Austria and in the UK. Ensuring a sufficient range of countries to reflect the different contexts in Europe was one of the selection criteria for interventions included in this study. This was accomplished to reflect qualitative evidence of early years interventions carried out in countries which were not represented in published literature [23]. Respondents from these countries described interventions being delivered within a context of insufficient children’s social and health service provision. These were delivered, in part, to bridge the gap of insufficient services to families mostly from disadvantaged backgrounds. Programmes provided activities to stimulate children’s learning through structured play and provided support and assistance for parents. In these, parents were actively involved in activities and respondents referred to long-lasting trust based relationships between staff and parents as one of the basis for the success of these programmes. These were open to the community; however, activities had a special focus on children and families with disadvantages, with the exception of the universal health visitor programme, which targeted the entire population.

Programmes included in the study were aimed at improving child development during early childhood before children entered preschool. Programmes aimed to involve parents in structured play related activities together with providing them with counselling, parenting classes and support. Evaluation studies published elsewhere [26] showed that Let’s Play in Tandem, a compensatory education programme which also involved parents and structured play, improved results tests of academic knowledge, receptive vocabulary, inhibitory control and school readiness. Strengthening parenting abilities helped them assist in their children’s learning and development. Interventions involved learning through play and were flexible to ensure parents’ participation. In like manner, The Incredible Years programme [27] which focuses on children’s social and emotional development involved parents and was delivered through play. It encouraged involvement to promote positive management skills and self - regulation. Other previous studies illustrated ways in which parenting activities delivered across income groups [8, 11] were not limited to cognitive gains, but also included physical, social, and emotional gains, all of which are determinants of health over the life course [12, 23, 28–30]. However, while focusing on parenting is important, it is also necessary to address the conditions of daily life which make positive parenting difficult. This requires policies aimed at children through an explicit, multi-dimensional and integrated strategy [15] and investment in reducing child poverty and improved living conditions [28, 29].

Our findings, concur with previous studies which described that an important aspect of early years programmes is establishing quality relationships between the deliverer and the recipient as well as ensuring that the recipients receive programmes relevant to their needs As described by the majority of staff, delivering an intervention - aimed at young children and their parents - effectively, entails recognising the knowledge and capacities of parents and involving them in the programme as key agents. Similarly, a qualitative evaluation carried out on the Healthy Schools programme showed that parental engagement was viewed by staff as a key factor [30]. The Childhood Development Initiative-Early Years care and education programme provided home visits and activities for parents based on their specific needs. Children receiving the service scored higher in rating scales and the Parents Plus Community Course improved children’s home learning environment [31]. Further studies found that providing a range of different levels of intensity according to need and tailoring to parents’ necessities and circumstances showed favourable outcomes in child behaviour. It also reduced abusive parenting styles, an example of this is the Positive Parenting Programme delivered in the UK and in Scotland [7, 31].

Programmes within this study were delivered by staff from different disciplines, some such as *Netzwerk Familie* were provided by a network of multidisciplinary professionals. A comprehensive range of services with the potential to improve child development were delivered by staff with different backgrounds. An previous study showed that Sure Start in the UK also collaborated with existing services and showed a multidisciplinary approach [32].

Beneficiaries referred to fear of being judged as a barrier as well as some reluctance towards the programmes. In addition, insufficient capacity and resources limited the number of children, families and/or mother attending the centres. The exception to this consensus was the health visitor programme in Hungary. Staff and parents put forward different views, while both groups agreed that limited available resources and space were a barrier, staff identified parents’ reluctance to attend some services and parents highlighted a bureaucratic approach by staff. Funding was described as a very important obstacle by staff working in programmes which were not funded by the government. Stigmatisation of users and/or showing some mistrust towards service providers and programmes may be common - particularly within a prevailing culture of low levels of service provision. Furthermore, the gap in service provision was accompanied in some areas by a reduced use of existing infrastructures and lack of intersectoral collaboration. Staff referred to knowledge, expertise and experience within the team as key enablers. Similarly, CDI Early Years, a child care and education programme which provides support to families living in an area of disadvantage is delivered by specialised staff. This was described as one of its pivotal elements [30]. Providing parents with support to engage with local area amenities and services and other families was also described by staff as an enabler for ensuring positive and less stressful parenting styles. Providing support in early stages helped ensure these were maintained. The Preparing for Life home-visiting programme, provided parents with support and showed positive outcomes: at 24 months children in the high treatment group showed stronger cognitive development and problem-solving skills. They showed fewer problem behaviours such as dysregulation, sleep problems, or clinically significant levels of internalising and externalising problems [12]. Likewise, establishing links with other local services and with the community has been described elsewhere as a key point for successful programme delivery [33]. As further studies showed, programmes which create bonds with the family home and establish links between it, the community, schools and other multiple stakeholders, present opportunities for multidisciplinary working and synergies for the delivery of child services [12].

Staff and users gave very similar accounts across programmes through their experiences, parents expressed a high level of satisfaction. This could be due to the fact that parents, who were facing adverse and very challenging circumstances, showed gratitude toward the provision of services which may have alleviated their situation and provided support. This could be reinforced with positive relationships with the staff who listened and did not judge, according to parents. Furthermore, parents were described by staff and themselves as being very overburdened. They may have avoided appearing overly critical or ungrateful with the programme fearing it may have led to discontinuing to receive services. In a similar way, staff who were aware of the limited services in their areas and the high level of disadvantage of families, may have seen any possible limitations of programmes as issues which could be improved rather than outright negative features.

Parents referred to feeling supported by non-judgmental staff. This could possibly be a key enabler not only for programme delivery but for other factors which were described in the interviews and focus groups as facilitating elements for successful delivery of programmes. These were establishing long lasting and trust based relationships, overcoming the fear of being judged as bad parents, being stigmatised or being referred to social services.

### Strengths and limitations of this study

The study had several limitations, the interviews and focus groups were performed by collaborating partners in each country, this may have contributed to less homogeneity in data collection. However, it enabled carrying these out in the informant’s native language and in the majority of instances, interviewers were involved in delivering the intervention and were therefore able to identify interviewees. Individual in-depth interviews and focus groups were used. The former enabled interviewing hard to reach informants who may have been reluctant to participate in focus groups and for senior staff. The latter provide valuable and rich information due to the dynamics of group discussion [34]. Interviewers from each collaborating institution provided University College London with summaries of the notes taken and recordings of sessions. These differed in length and detail. The limitations however were mitigated by the fact that UCL provided a common template and guide for third parties to carry out the case studies. These included guidance for individual interviews, focus groups and for providing socioeconomic indicators. Furthermore, Joana Morrison and Peter Goldblatt visited the intervention sites and discussed data collection issues with all third parties and provided advisement and assistance throughout the whole process over the phone and via email.

## Conclusions

Based on the accounts of both parents and staff, the establishment of a long term trust based relationships is a key enabler in the delivery of programmes and services, especially to socially isolated or hard to reach families and children. Programmes described by staff as being successful in providing support and building on parent’s capacities, delivered services that adapted to the needs and circumstances of parents and their children providing activities carried out by teams which included social service workers, doctors, and staff with early years training. Adapting to and understanding the families’ circumstances and involving parents was seen by staff as necessary to contribute to empowering parents and developing their own skills and in turn assist in their children’s learning. According to staff, this also had a positive effect on children.

## Recommendations and policy implications

It is important to provide access to a comprehensive range of quality early year services to reduce inequalities during the early development of children, especially for those from disadvantaged backgrounds. Services should be tailored to social and economic need. It is important to recognise the knowledge and capacities of parents if interventions aimed at young children and their parents are to be delivered effectively and sustained over time. To ensure that parents have an active involvement in early years programmes, they should be empowered and receive support and information to understand and contribute to the optimal development of their children. Existing ECD institutions and structures should be strengthened to promote working between the social, education and health sectors. The recognition, representation and funding of ECD in all areas of work and policy should be enhanced through high-level leadership. This includes promoting support for children who are deprived or vulnerable. Programmes delivered in families’ homes and in accessible centres should be evaluated so as to compare outcomes when using one or other of these settings or a combination of the two.
